# Growth and Toxin Production of *Gambierdiscus* spp. Can Be Regulated by Quorum-Sensing Bacteria

**DOI:** 10.3390/toxins10070257

**Published:** 2018-06-22

**Authors:** Bo Wang, Mimi Yao, Jin Zhou, Shangjin Tan, Hui Jin, Feng Zhang, Yim Ling Mak, Jiajun Wu, Leo Lai Chan, Zhonghua Cai

**Affiliations:** 1 School of Life Science, Tsinghua University, Beijing 100084, China; cuhkbobwong@gmail.com (B.W.); shangjin-tan@163.com (S.T.); jinhui0127@126.com (H.J.); 2 Center for Microalgal Biotechnology and Biofuels, Institute of Hydrobiology, Chinese Academy of Sciences, Wuhan 430070, China; mimiyao@ihb.ac.cn; 3 Shenzhen Public Platform of Screening & Application of Marine Microbial Resources, Graduate School at Shenzhen, Tsinghua University, Shenzhen 518055, China; zhou.jin@sz.tsinghua.edu.cn; 4 State Key Laboratory in Marine Pollution, City University of Hong Kong, Hong Kong 999077, China; zhang_feng1220@163.com (F.Z.); maggieylmak@yahoo.com.hk (Y.L.M.); jiajun@hkc.edu.cn (J.W.); Leochen@hkc.edu.cn (L.L.C.); 5 Shenzhen Key Laboratory for the Sustainable Use of Marine Biodiversity, Research Centre for the Oceans and Human Health, City University of Hong Kong Shenzhen Research Institute, Shenzhen 518057, China

**Keywords:** Ciguatera fish poisoning, *Gambierdiscus*, quorum sensing, algal–bacterial relationship

## Abstract

*Gambierdiscus* spp. are the major culprit responsible for global ciguatera fish poisoning (CFP). At present, the effects of microbiological factors on algal proliferation and toxin production are poorly understood. To evaluate the regulatory roles of quorum-sensing (QS) bacteria in the physiology of *Gambierdiscus*, co-culture experiments with screened QS strains were conducted in this study. Except for the growth-inhibiting effect from the strain *Marinobacter hydrocarbonoclasticus*, the algal host generally displayed much higher growth potential and toxin production ability with the existence of QS strains. In addition, *Bacillus anthracis* particularly exhibited a broad-spectrum growth enhancement effect on various *Gambierdiscus* types, as well as a remarkable influence on algal toxicity. The variations of algal physiological status, including growth rate, chlorophyll content, and responsive behaviors, are potential reasons for the observed positive or negative affection. This study suggests that QS bacteria regulate the algal growth and toxin production. Based on the evidence, we further speculate that QS bacteria may contribute to the site-specific distribution of CFP risk through regulating the algal host biomass and toxicity.

## 1. Introduction

Public health around the world is threatened by the ciguatera fish poisoning (CFP), which is a serious syndrome that is caused by the ingestion of tropical and subtropical reef fish contaminated with lipophilic ciguatoxins (CTXs) [1]. Any toxicity that exceeding 0.31 ng/g P-CTX-3C equivalent of flesh would cause signs of intoxication including neurological, gastrointestinal, and cardiovascular dysfunctions [2,3]. Different groups of ciguatoxin analogues have been identified according to their geographic distribution, including the C-CTXs from the Caribbean Sea, P-CTXs from the Pacific Ocean, and I-CTXs from the Indian Ocean [4]. CTXs can accumulate in the liver to life-threatening levels and lead to death [5]. With the expanding of the international tropical fish trade, CFP has spread throughout the world, including places such as Hong Kong [6], Southeast Asia [7], Australia [8], and America [9]. By the end of the 20th century, more than 50,000 people each year suffered from CFP, and the global CFP incidences continue to increase [10]. In 2002, 464 CFP cases per 10,000 people were recorded in French Polynesian Raivavae Island [11]. Alongside the direct impact on human health, CPF also caused huge economic loss to the marine fishing industry. In America alone, the annual loss is estimated to $22 million [12].

Several toxic benthic dinoflagellate belonging to the genus *Gambierdiscus*, which produce CTX intermediates (52-epi-54-deoxy-CTX-1B and 54-deoxy-CTX-1B) and gambiertoxins, are confirmed as the primary source of CTXs [13,14]. The *Gambierdiscus* could usually be found in microbial biofilms attached on the macroalgal surface [15]. The macroalgae are the food source of the herbivorous coral reef fishes, which could be further ingested by carnivorous fishes. In this process, toxin precursors and their intermediates are transferred into the food chain, and then biotransformed to the relatively oxidized CTXs, which are the more toxic final products [16]. Therefore, the toxin contamination in the fish and the resulting CFP risk are highly related to the total biomass and toxicity of the ingested *Gambierdiscus*. However, the CFP risk often exhibits site-specific spatial distribution characteristic [17]. As a typical example, the blue-spotted grouper (*Cephalopholis argus*), undulated moray (*Gymnothorax undulatus*) and yellow-edged moray (*Gymnothorax flavimarginatus*) collected near the western coast of Marakei Island, Republic of Kiribati, are at least 30 times more toxic than those collected from adjacent regions [18]. Interestingly, the food source composition of fish is different among regions with different CFP risks. According to an isotopic tracing study [19], the ingested biofilms account for a prominent proportion (16.4–29.9%) in the food source of fishes from ciguatoxic sampling sites, while the fishes from the reference site have a more balanced diet. As the major components in the bacteria/algal biofilm [20], it is reasonable to infer that the bacterial community and *Gambierdiscus* correlate with the CFP risk.

Previously, the *Gambierdiscus* proliferation and toxicity have been reportedly influenced by nutrients, temperature, light, and salinity conditions [21,22,23]. However, the function of the microecological process has received little attention; limited studies have concerned the function of epiphytic microbial communities inhabiting on algal surfaces [24,25,26]. In fact, bacteria, in symbiotic relationships with algae, form the basic and active link in the phycosphere by extensively participating in substance cycling, oxidation-reduction activities, and the regulation of algal physiological behavior [27]. Many life processes including growth, defense, and toxin production, are mediated by physiochemical interactions at the individual, population, and community levels [28]. In addition, various community behaviors of heterotrophic bacteria have been identified in algal–bacterial symbionts through the abundant infochemicals in the phycosphere [28,29,30]. As a typical process involving infochemicals, quorum sensing (QS) controls community behaviors by the population density-dependent diffusible signaling molecules, typically the N-acyl homoserine lactone (AHL) [31,32,33]. It has been reported that QS signals from algal-associated epiphytic or endophytic bacterial isolates could regulate a variety of bacterial behaviors such as biofilm formation, phenotype adaptation, exopolysaccharide production, zoospore settlement, carpospore liberation, virulence production, and motility, most of which are essential for the successful establishment of a symbiotic relationship with a eukaryotic host [34,35,36,37,38]. During the above processes, the bacteria and algae could interact in different ways, and the phycosphere environment could be significantly adjusted. Therefore, investigating the function of QS microbes in the *Gambierdiscus* phycosphere will help to better understand their roles in algal physiology and the potential influences on the distribution of CFP risks.

Inspired by previous reports, it is reasonable to speculate that through algal–microbial interactions, QS microbes play an important role in regulating the *Gambierdiscus* physiology, such as growth and toxin production. However, limited evidence is available to support this hypothesis at present. Therefore, in this study, we isolated and screened several AHL-producing strains from the Marakei Island of the Republic of Kiribati, and then investigated their effects on the growth and toxin production of several *Gambierdiscus* strains by co-culture experiments. The aim of this study is to investigate whether and how the signaling bacteria could influence the growth potential and toxicogenic behavior of *Gambierdiscus*, and have a better understanding of the reasons leading to the site-specific spatial distribution phenomenon of CFP.

## 2. Results

### 2.1. QS Bacteria Screening

In the 96-well microtiter-plate test (Figure S1a), more than 900 bacterial isolates from sampling sites were screened for AHL production, and 32 isolates were identified as potential AHL-producing candidates. After the removal of false positives, nine bacterial strains that showed positive signals and blue color zones with the bioreporter strain A136 remained (Figure S1b). The identities of these QS candidates were determined by 16S rDNA gene sequencing and the Basic Local Alignment Search Tool (BLAST) alignment. The closest match of each corresponding bacterial species was selected and listed in Table 1. In addition, according to the taxonomic distribution (Genus level) of the four sampling sites (Figure S2), the corresponding Genus distribution of the screened QS bacteria was listed in Table S1. The 16S rRNA phylogenetic tree of the screened bacteria was showed in Figure S3.

### 2.2. Effects of QS Bacteria on Algal Growth

**Figure 1:**
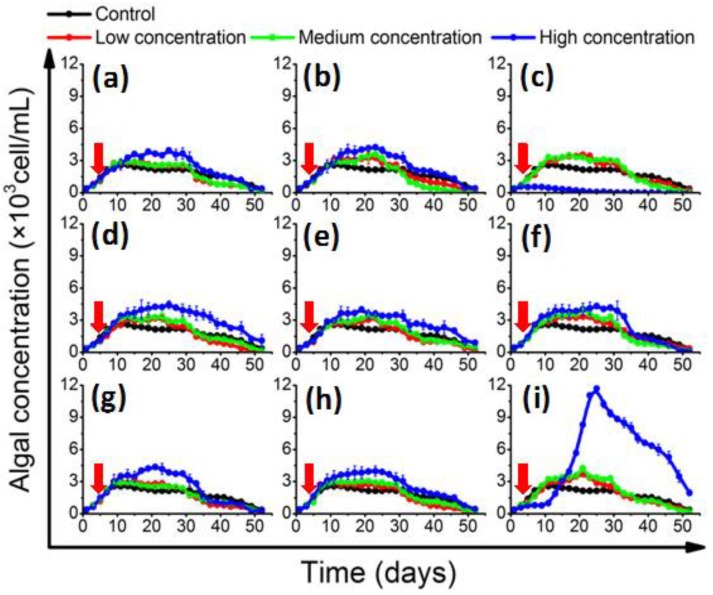
Growth of 1022M2C12 after co-culture with different concentration of (**a**) *Vibrio* sp.; (**b**) *A. macleodii*; (**c**) *M. hydrocarbonoclasticus*; (**d**) *Thalassospira* sp.; (**e**) *P. aeruginosa*; (**f**) *V. maritimus*; (**g**) *P. vermicola*; (**h**) *M. stanieri*; and (**i**) *B. anthracis*. Low concentration: 5 × 10^3^ cells/mL. Medium concentration: 5 × 10^4^ cells/mL. High concentration: 5 × 10^5^ cells/mL. Red arrow: time point of bacteria addition.

In the log-phase co-culture experiment, the algal concentration and growth rate were monitored (supplementary material). In the control group, the 1022M2C12 (*Gambierdiscus* sp. type 5, Table S2) reached stationary phase on the 10th day; the concentration was about 2500 cells/mL (Figure 1). The growth rate reached about 0.369 divisions day^−1^ (Table S4), which was consistent with a previous report regarding optimum culture conditions [3,39]. With the adding of AHL-producing bacteria, the algae in the experimental groups generally exhibited a higher cell yield in a bacterial concentration-dependent pattern. The algal concentration in the presence of low- and medium-concentration bacteria were moderately higher than that of the control, while it was significantly (*p* < 0.05) increased in high-dose bacterial co-cultures, reaching 4000 cells/mL at the stationary phase. It is worth noting that algal proliferation was initially inhibited by the addition of high-concentration (5 × 10^5^ cells/mL) *B. anthracis*, and then increased dramatically (Figure 1i). The bloom-like behavior continued until the algal density peaked at approximately 12,000 cells/mL on the 25th day. On the contrary, the algal growth was significantly (*p* < 0.05) inhibited by a high concentration of *M. hydrocarbonoclasticus* (Figure 1c). The growth rate decreased to 0.056 ± 0.006 divisions per day^−1^ (Table S4), and no recovery of algal growth was observed.

**Figure 2:**
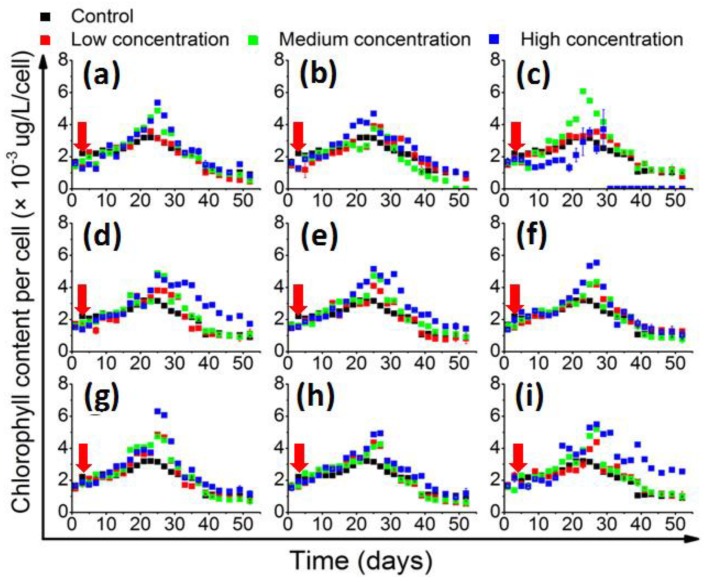
The change of in vivo chlorophyll content (chlorophyll content per cell) of 1022M2C12 co-cultured with (**a**) *Vibrio* sp.; (**b**) *A. macleodii*; (**c**) *M. hydrocarbonoclasticus*; (**d**) *Thalassospira* sp.; (**e**) *P. aeruginosa*; (**f**) *V. maritimus*; (**g**) *P. vermicola*; (**h**) *M. stanieri*; and (**i**) *B. anthracis*. Low concentration: 5 × 10^3^ cells/mL. Medium concentration: 5 × 10^4^ cells/mL. High concentration: 5 × 10^5^ cells/mL. Red arrow: time point of bacteria addition.

The in vivo chlorophyll quantification results (Figure 2) showed that the cellular chlorophyll content generally accumulated slowly in the beginning, and then accelerated and culminated in the middle of the stationary phase. In the control group, the chlorophyll content peaked at 3.1 × 10^3^ μg/L/cell on the 22nd day. Most of the QS bacteria that significantly enhanced the growth of 1022M2C12 were also effective in increasing the cellular chlorophyll content. However, the in vivo chlorophyll content was significantly (*p* < 0.05) promoted by medium-concentration *M. hydrocarbonoclasticus* and reached 6.0 × 10^3^ μg/L/cell, while the high concentration of the bacteria had decreased (Figure 2c). The high-concentration *P. vermicola* also stimulated the cellular chlorophyll content to 6.2 × 10^3^ μg/L/cell, which was significantly higher than that of the other experimental groups.

### 2.3. The Growth Promotion Principle of QS Bacteria

To dissect the principle of this growth promotion, *B. anthracis* was applied as a representative strain for further study, owing to its significant growth promoting potential. We assessed (1) the effective regulatory phase of adding the QS bacteria; (2) the existence of potential intracellular or extracellular bioactive substances; and (3) the species-specific growth enhancement effect of QS bacteria to the genus *Gambierdiscus*.

**Figure 3:**
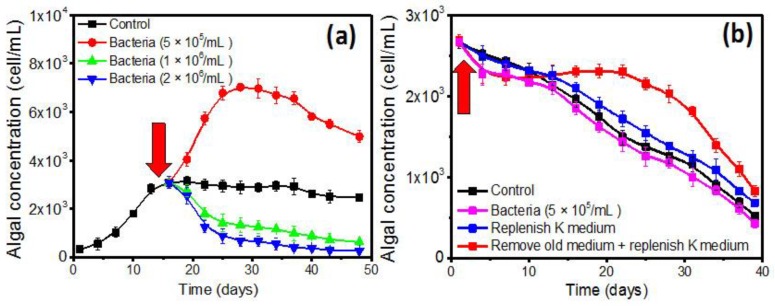
Growth of 1022M2C12 in (**a**) the stationary phase; and (**b**) the decline phase after co-culture with *B. anthracis*, nutrient supplement, and removal of secondary metabolites, respectively. Red arrow: time point of addition.

As shown in Figure 3a, at the beginning of the stationary phase, the algal growth was rapidly affected by the addition of bacterial suspensions. The added 5 × 10^5^ cells/L *B. anthracis* exhibited a significant enhancement effect, leading to an additional 0.1496 ± 0.0223 divisions day^−1^. The algal yield reached 7000 cells/mL. However, an inhibitory effect emerged after further increasing the concentration of added bacteria. The result indicated the existence of a threshold value of the added bacteria. The effect of QS bacteria in the decline phase was also evaluated; however, no enhancement in algal growth was observed (Figure 3b). For comparison, we assessed the effects of nutrient supplementation and secondary metabolite removal. Only a slight stimulation (0.0186 ± 0.0103 divisions day^−1^) and extended lag phase in the secondary metabolite removal group were observed (Figure 3b).

**Figure 4:**
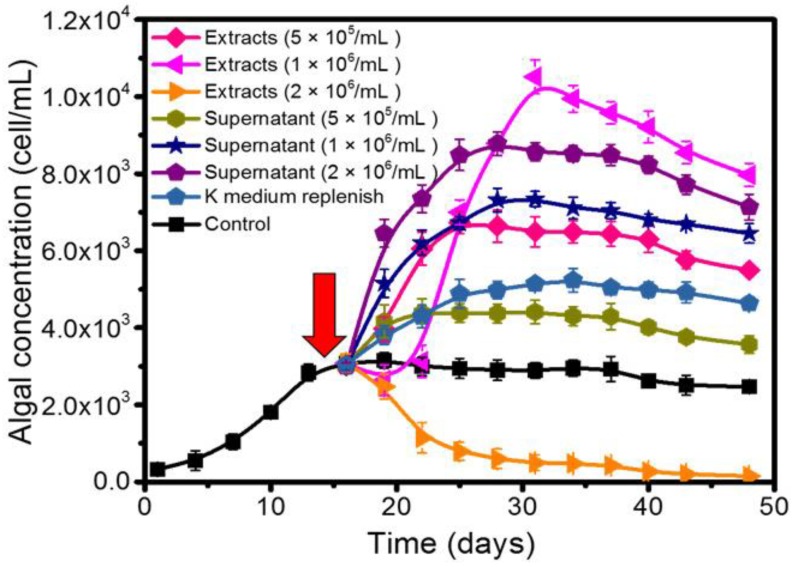
Effect of bacterial extracts, supernatants, and nutrient supplements on the growth of 1022M2C12 at an early stationary phase. Red arrow: time point of addition.

Next, the existence of potential intracellular (in bacterial extracts) and extracellular (in supernatants) bioactive substances was investigated. Besides, the effect of nutrient supplementation was also investigated for comparison study. Consistent with the stationary phase co-culture experiment (Figure 3a), a rapid growth change of 1022M2C12 was observed after the addition of both bacterial extracts and supernatants (Figure 4). The extracts from 1 × 10^6^ cells/mL bacterial culture exhibited the strongest enhancement effect, leading to an additional 0.1811 ± 0.0129 divisions day^−1^ to the algal growth rate. The yield was improved to approximately 1.0 × 10^4^ cells/mL, which was four times that of the control group. The algal proliferation was also remarkably stimulated by the supernatants from 2 × 10^6^ cells/mL bacterial culture (Figure 4), resulting in an additional 0.216 ± 0.018 divisions day^-1^ growth rate. It is worth noting that although a prolonged log phase was observed after the addition of the fresh nutrients, the effect was not as pronounced as that of most of the other extract and supernatant groups.

**Figure 5:**
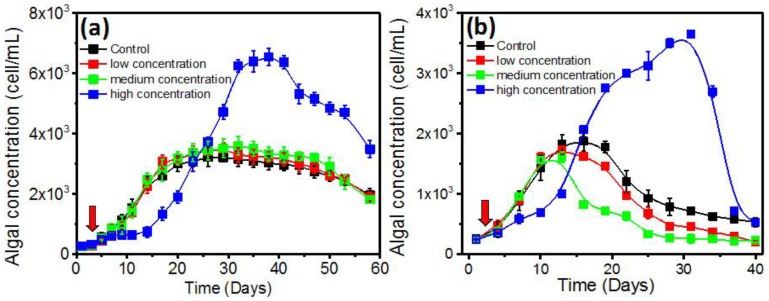
Effect of *Bacillus anthracis* on the growth of (**a**) 1112M1M03 (*Gambierdiscus* sp. type 6) and (**b**) 1021M1DC4 (*Gambierdiscus* sp. type 6). Red arrow: time point of bacteria addition. Low concentration: 5 × 10^3^ cells/mL bacteria. Medium concentration: 5 × 10^4^ cells/mL bacteria. High concentration: 5 × 10^5^ cells/mL bacteria. Red arrow: time point of bacteria addition.

To investigate whether the growth enhancement effect of QS bacteria is species-specific to the genus *Gambierdiscus*, the growth of two additional *Gambierdiscus* strains (1112M1M03 and 1021M1DC4) in the presence of *B. anthracis* were also studied. Figure 5 revealed that the high-concentration bacteria (5 × 10^5^ cells/mL) could also promote the growth of the two strains, while the extent and pattern of this effect differs between species. The highest yield that 1112M1M03 (0.02 pg P-CTX-1/cell) and 1021M1DC4 (no toxicity) reached were 6.6 × 10^3^ (Figure 5a) and 3.6 × 10^3^ cells/mL (Figure 5b), respectively.

### 2.4. Influence of QS Bacteria on Algal Toxicity

**Figure 6:**
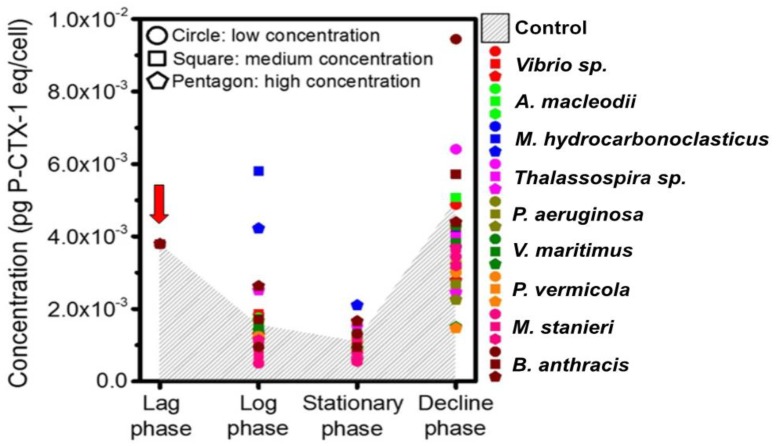
Toxicity of 1022M2C12 (*Gambierdiscus* sp. type 5) in different growth phases after co-culture with the screened quorum-sensing (QS) bacteria. Low concentration: 5 × 10^3^ cells/mL bacteria. Medium concentration: 5 × 10^4^ cells/mL bacteria. High concentration: 5 × 10^5^ cells/mL bacteria. Red arrow: time point of bacteria addition.

The influence of all the screened AHL-producing bacteria on the toxin production of 1022M2C12 was investigated. Generally, the toxicity of the control group periodically declined during the lag phase and improved in the decline phase (Figure 6). The algal toxicities in the control group from the lag phase to the final decline phase were 3.797, 1.553, 1.092 and 4.975 × 10^−3^ pg P-CTX-1 eq/cell, respectively. In the log and stationary phases, the algal toxicity was most strongly promoted by *M. hydrocarbonoclasticus*, reaching 5.804 and 2.103 × 10^−3^ pg P-CTX-1 eq/cell, respectively. Meanwhile, *M. stanieri* significantly (*p* < 0.05) reduced the toxicity to 0.4 × 10^−3^ pg P-CTX-1 eq/cell at the log and stationary phases. In the decline phase, the low concentration of *B. anthracis* improved the algal toxicity to a remarkable 1.0 × 10^−2^ pg P-CTX-1 eq/cell, which was significantly (*p* < 0.05) higher than the control. In contrast, *V. maritimus* and *P. vermicola* decreased the algal toxicity to 2.0 × 10^−3^ pg P-CTX-1 eq/cell.

## 3. Discussion

Predicting the risk of CFP is difficult, because the abundance and toxicity of genus *Gambierdiscus* varies between different areas, and the digestion behaviors and metabolic pathways of fish may also affect the fate of CTX biotransformation [40]. However, it is plausible that the ingested biomass and toxicity of *Gambierdiscus* are the decisive factors that affect the CFP risk according to the food chain theory [14]. Diverse populations of microorganisms exist in phycosphere environments, and the algae–bacteria interactions mutually affect their physiology and alter the flux of matter and signaling compounds, shaping the diversity of the ecosystem [30]. Previous studies have shown that the algae could be greatly affected by the symbiotic bacteria, and those interactions were mediated through the production and exchange of infochemicals [30]. Therefore, understanding signaling strains may shed light on the effects of algae-associated microbial communities on the native host. Based on the ecological importance of signaling microbes, this study sought to interpret the modulatory functions of QS bacteria in algal growth and toxin production from a microbial–ecological perspective.

**Figure 7:**
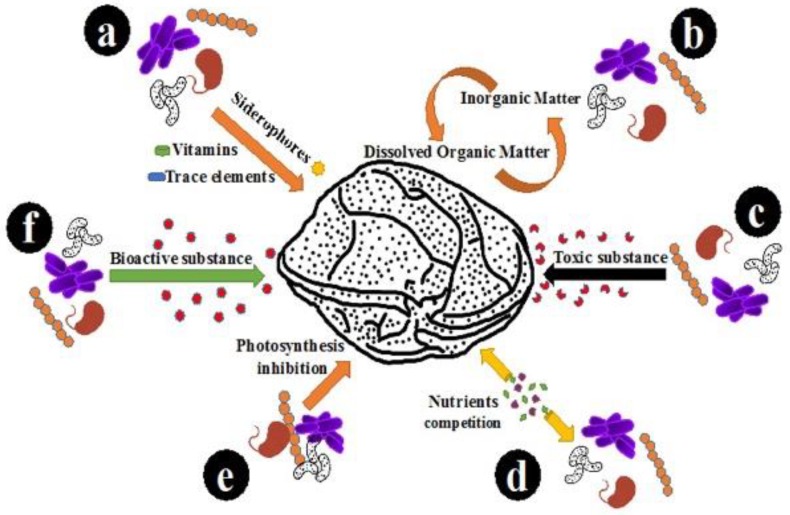
Scheme of the interactions between QS bacteria and *Gambierdiscus*.

The co-culture experiments were prepared in xenic condition, because the benthic *Gambierdiscus* commonly live with associated bacterial flora [20], and the symbiotic relationship is potentially important for the life process of the algae. Besides, no differences have been previously found in the growth and toxicity of *Gambierdiscus* strains assayed in axenic versus xenic culture conditions [3,41]. Adding antibiotics may also bring an additional impact factor to the co-culture experiment. The results showed that the screened AHL-producing strains can affect the growth of 1022M2C12 (*Gambierdiscus* sp. type 5, Figure 1). The algal yield generally increased with the addition of signaling bacteria, and the effect is generally better than that of the reported optimum environmental conditions (i.e., temperature, salinity, and irradiance) on the growth of several *Gambierdiscus* spp. [23], suggesting that algae might acquire beneficial nutrients from the QS microbes or their metabolic products. In concordance with previous work, which showed that *Alteromonas* sp. could stimulate the growth of *Gambierdiscus toxicus* by providing “public goods” to its host [24], an *Alteromonas* strain (*A. macleodii*) in this study also exhibited the similar growth stimulatory effect. The generally increased in vivo chlorophyll contents (Figure 2) are also good evidence indicating the existence of sufficient energy sources for chlorophyll accumulation. The accumulated chlorophyll content could lead to stronger photosynthesis ability and higher biomass accumulation for cell division. The QS bacteria might directly mediate this process by acting as sources (Figure 7, part a) for siderophore production [42], trace elements adsorption, and vitamin supply [43,44], or indirectly by re-mineralizing the dissolved organic matter (DOM) excreted from phytoplankton (Figure 7, part b). The process can facilitate the microbial recycling of fresh organic matter, and thus strengthen the role of the microbial loop in the phycosphere environment [45].

In addition to the enhancement effects, a clear algal growth-inhibitory role was observed from the high-concentration of *M. hydrocarbonoclasticus* (Figure 1c), exhibiting a distinct negative effect to the physiology of *Gambierdiscus*. A similar phenomenon could be observed from *Gambierdiscus toxicus*, which was significantly inhibited by *Flavorbacterium* sp. strain C1 [24]. This effect could be potentially due to bioactive substances released from the bacterial cells that are toxic to the algae (Figure 7, part c), resulting in a lethal effect when exceeding a threshold value. Alternatively, growth inhibition could be the result of nutrient competition between the bacteria and algae (Figure 7, part d) [46]. In addition to these potential scenarios, it is worth noting that the in vivo chlorophyll content of 1022M2C12 co-cultured with a high concentration of *M. hydrocarbonoclasticus* kept accumulating until the 30th day (Figure 2c), while not leading to cell growth (Figure 1c). Besides, the higher content of in vivo chlorophyll in the algae cultured with a medium concentration of *M. hydrocarbonoclasticus* did not led to additional algal accumulation compared with the low bacterial concentration group (Figure 1c). The result indicates that the photosynthesis process was inhibited. Therefore, a reasonable explanation for the growth inhibition is that a high concentration of *M. hydrocarbonoclasticus* might have affected the light adsorption and the photosynthesis process by direct or indirect interactions (e.g., inhibiting the function of photosystem), resulting in the inhibition of algal growth (Figure 7, part e). To confirm the speculation, further investigations are needed. From the current results, we proposed that *M. hydrocarbonoclasticus* might play a crucial role in the decline phase of *Gambierdiscus*.

Compared to the slow and absent algal responses in the log and decline phases (Figure 1 and Figure 3b, respectively), the expansion of algal biomass in the early stationary phase was rapid and distinctive (Figure 3a). It reveals that the stimulatory role is most prominent during the stationary phase. Interestingly, the results indicate that similar to the density-dependent quorum sensing behavior of bacteria, the responsive ability of algae correlated with algal density. Generally, the physiology of algae will reach the optimum state and highest stability in the early stationary phase. It is reasonable that a high concentration of high-quality algae might allow for the prompt response to extraneous stimulation and better buffering of adverse conditions. In the co-culture experiment at the decline phase, the rebound in the secondary metabolite removal group demonstrated that the algal physiology was seriously affected by the deteriorated environment, leading to a lack of enhancement by QS bacteria in the decline phase (Figure 3b). The negative result illustrated that the effect of QS bacteria on *Gambierdiscus* is affected by the abiotic factors, biotic status, and physiological conditions of the algae.

The stimulatory effect of bacterial extracts and fermentation supernatants were also studied in order to identify and locate the potential bioactive substances. As illustrated in Figure 4, most of the experimental groups strongly promoted algal growth, and the performance was much higher than that of the nutrient supplementation. The result indicated that the extract and supernatant of the bacterial culture may have either provided higher nutrient concentration, or released growth-promoting bioactive substances to the algae (Figure 7, part f). The rapid response of algal growth revealed that the potential substance might act as a key growth factor that was used up at the end of the log phase. Therefore, it is suspected that either sufficient nutrients may exist in the bacterial extract and culture supernatant, or that bioactive substances that effectively stimulate algal growth were synthesized and released by the QS bacterial cells. However, the reason for the inhibitory effects of higher concentrations of living bacteria (Figure 3a) and extracts (Figure 4) is still unclear. Although the nutrient competition and growth inhibition by the bacterial endotoxins [47] could offer plausible explanations, enough evidence does not currently exist to support the hypothesis. Detailed information is essential for future study.

The growth stimulation effect on two additional *Gambierdiscus* strains with different toxicities illustrated that the QS bacteria *B. anthracis* possesses a broad-spectrum effect on the genus *Gambierdiscus*. Considering the remarkable bloom-like behavior of the three strains during co-culture experiments, this specific bacterium is likely to play an important role during the proliferation process of genus *Gambierdiscus*. More importantly, the different levels of growth stimulation among the three strains demonstrated that QS bacteria may specifically affect the concentration of *Gambierdiscus* with different toxicities. Thus, based on the evidence obtained from this study, we speculate that the QS bacteria act as a fundamental factor that regulates the site-specific spatial distribution of CFP risk.

The periodic variation of algal toxicity has been a topic for discussion for long time. It is widely believed that increasing toxicity is a self-protective strategy that empowers the algae with a higher competing ability in a deteriorative environment, which is the result of nutrient exhaustion and harmful metabolite accumulation in the late stationary phase. In an improved nutritional environment for algal survival, the acquired energy was preferentially utilized for cell division instead of toxin production. Therefore, the generally decreased toxicity in most groups supported the existence of a nutrient-transferring relationship between QS bacteria and *Gambierdiscus* (Figure 7, part b). However, the presence of multiple species-specific bacteria could also induce algal responses such as toxin production for self-protection [48]. Consistent with earlier speculations, the toxin production of algae was significantly enhanced by *M. hydrocarbonoclasticus* during the stationary phase (Figure 6), indicating that the algae were under intense pressure from the bacteria through altering the nutrient availability or interacting by harmful bioactive substances. In addition, distinct concentration-dependent behaviors were observed from *B. anthracis*, which promoted the algal toxicity at low concentrations (Figure 6) and enhanced the algal growth at high concentrations (Figure 1i). As a QS bacterium, when the community achieved certain density, the expression of specific genes could be activated and resulted in different microbial behaviors [31]. Therefore, the QS-regulated behavior is highly suspected to lead to different effects on *Gambierdiscus*. In future study, more efforts should be made to identify the substance and confirm the hypothesis. Additionally, it is worth noting that several *Gambierdiscus* species are non-toxic; the toxin-regulating effect of the QS strains used in this study should be further checked on those non-toxic species to investigate whether a universal toxicity regulation in QS bacteria exists toward *Gambierdiscus*.

## 4. Conclusions

In this study, we identified several QS bacterial strains that could affect the growth and toxin production of *Gambierdiscus*. Both positive and negative regulatory effects were found to occur in algal–bacterial symbionts, and the bioactive effects were observed from bacterial extracts and supernatant fermentation. Among the screened QS bacteria, the bacteria *B. anthracis* exhibited the most potent broad-spectrum stimulatory effects, with various extents on different *Gambierdiscus* strains. On the contrary, the *M. hydrocarbonoclasticus* appears to play an important role in the decline of algae. Nutrient transferring, sources competition, toxic substance releasing, and photosynthesis inhibition are suspected to be involved in the algal–microbial interaction process. From a quorum-sensing perspective, the present work indicates that the algal–microbial relationship is highly complex and worthy of deep investigations. In addition, we found by affecting the growth and toxicity of the *Gambierdiscus* that the QS bacteria probably play an important role in the site-specific distribution of the CFP risk. Taken together, this study tells us that understanding the ecological roles of the QS phenomenon in the holobiont-associated community is beneficial for expanding our knowledge of the algal–bacterial relationship. However, a systematic investigation is still needed for determining the underlying mechanisms and finding an effectively way to predict and protect people from CFP risk.

## 5. Materials and Methods

### 5.1. Sample Collection and Algal Culture

The natural samples were scratched from the surface of red algae, dead coral, and seaweed from four sampling sites around Marakei Island, Republic of Kiribati, including M1 (2°01.394′ N, 173°15.383′ E), M2 (1°59.879′ N, 173°15.032′ E), M3 (1°58.246′ N, 173°16.268′ E), and M4 (2°00.150′ N, 173°18.010′ E). The fresh samples were immediately processed for algal isolation and QS bacteria screening. Three Pacific ribotype strains of *Gambierdiscus* were isolated, and the species information were identified through *Gambierdiscus* phylogenetic analyses [49], including 1022M2C12 (*Gambierdiscus* sp. type 5), 1112M1M03 (*Gambierdiscus* sp. type 6), and 1021M1DC4 (*Gambierdiscus* sp. type 6) (Table S2). The algal cultures were maintained in 225 cm^2^ angled neck cell culture flasks (Corning, NY, USA) in filtered seawater (salinity of 31.0 parts per thousand) supplemented with sterilized 100× stock solution of K medium [50]. To simulate the local environmental conditions of Marakei, all of the algal strains were cultured in a 26 °C incubator (LM-570RD, Yiheng, Shanghai, China) under 55-μmol photons m^−2^ s^−1^ cool-white fluorescent illumination with a 12 h:12 h light–dark cycle.

### 5.2. Bacterial Isolation and AHL-Producer Identification

The fresh samples were 100-fold diluted in sterilized seawater and spread on Zobell 2216E solid agar plates [51]. The plates were incubated at 25 °C for 48 h. The inoculation needles were used for picking bacterial colonies and transferring into 1 mL of autoclaved Zobell 2216E broth. After 12 h of incubation, all of the isolated samples were stored at −80 °C in 15% glycerol. Then, the bacterial isolates were screened for AHL production by using the indicator strain *Agrobacterium tumefaciens* A136. A fast liquid screening method in 96-well microtiter plates (Figure S1a) was developed based on the traditional method conducted on agar plates [52]. The bioassay was conducted as follows. All of the isolated samples were cultured in 1 mL of liquid Zobell 2216E medium overnight at 25 °C, and the indicator strain A136 was pre-cultured in liquid LB medium at 30 °C to stationary phase. Aliquots of 100-µL A136 and 100-µL cultured isolated samples were mixed in the 96-well microtiter plates, and 5 µL of X-Gal (40 μg/mL) were added into each well for colorimetric screening. The presence of a blue color after 24 h of incubation indicated candidate AHL-producing strains. The *Pseudomonas aeruginosa* PAO1, *Escherichia coli* DH5α, and ultrapure water were used as the positive control, negative control, and blank control, respectively. To conduct a more stringent second screen, 10 μL of the potential AHL-positive bacterial strains were pipetted onto 5-mm diameter circle filter paper on solid 2216E plates, and then supplemented with 10 μL of A136 and 5 µL of X-Gal (40 μg/mL), and incubated overnight at 30 °C. The visible blue pigmentation samples after incubation were retained (Figure S1b). To eliminate the false-positive results, the same method was conducted on the retained strains without the addition of A136, and the samples that still displayed a blue color were eliminated.

The genomic DNA of all of the screened AHL-producing bacterial strains were extracted using the StarPrep Bacterial DNA Kit (Gene Star, Beijing, China) and purified using the StarPrep PCR&DNA Fragment Purification Kit (Gene Star, Beijing, China) following the manufacturer’s instructions. The bacterial 16S rRNA gene (position 27-1492) was amplified by polymerase chain reaction (PCR) using universal 16s 27F/1492R primers (27F: 5′-AGAGTTTGATCATGGCTCAG-3′; 1492R:5′-TACGGYTACCTTGTTACGACTT-3′). Then, the obtained 16S rDNA were sent to the Beijing Genomics Institute (BGI, Shenzhen, China) for sequencing. The obtained sequences (Table S3) were aligned to the National Center for Biotechnology Information (NCBI) databases using the Microbial Nucleotide BLAST (basic local alignment search tool) to find the closest match of corresponding bacterial species. The MEGA 7 software was used for constructing the 16S rRNA phylogenetic tree of screened bacteria (Figure S3). The sequences of all of the screened QS bacteria were uploaded to the GenBank; the accession numbers are KY777596-KY777604. In addition, 100-mL aliquots of seawater samples from the four sampling sites were used to extract the environmental microbial genomics using the DNA Isolation Kit (Power Water 14900-S, Mo Bio, Carlsbad, CA, USA). The 16s rDNA V6 area (967F/1046R) of the obtained DNA samples were further sequenced by the Beijing Genomics Institute (BGI, Shenzhen, China). The four sampling sites’ taxonomic distribution at the genus level and the corresponding genus distribution of the screened QS bacteria were investigated.

### 5.3. Co-Culture Experiment at Early Log Phase

The 1022M2C12 (*Gambierdiscus* sp. type 5, 0.004 pg P-CTX-1/cell) was used to evaluate the growth effects of QS bacteria challenge in co-culture experiments. The algae in the early log phase (about 5 × 10^2^ cells/mL) was used for the co-culture experiments. The screened AHL-producing bacterial strains were pre-cultured in Zobell 2216E broth at 25 °C for 24 h. Prior to the experiment, bacterial densities were determined by a flow cytometer (BD Accuri C6, BD Biosciences, Billerica, MA, USA) after Syto9 staining. Different concentrations of bacterial suspensions (pre-washed by ultrapure water) were added to 500 mL of algal culture without antibiotic treatment. The final concentration of added bacterial suspension was set to low (5 × 10^3^ cells/mL), medium (5 × 10^4^ cells/mL), and high (5 × 10^5^ cells/mL) concentration levels, respectively. Strict sterile manipulation was carried out to avoid contamination during the co-culture experiments. The algal concentration was monitored every three days in triplicate using a 100-grid algal counting plate under an optical microscope (E200, Nikon, Tokyo, Japan). Besides, the growth rates (divisions day^−1^) of all of the experimental groups were calculated (supplementary material). To monitor the in vivo chlorophyll content (chlorophyll content per cell), 5-mL aliquots of algal samples were collected every three days during the co-culture experiment, and then added into a 10 × 10 mm quartz cuvette for measuring. The total chlorophyll content was determined using the total Chl function of a Phytoplankton Analyzer (PHYTO-PAM, Walz, Germany) in triplicate [53]. During the measuring, four LED with 470-nm, 520-nm, 645-nm, and 665-nm emission wavelengths were applied for the excitation of chlorophyll fluorescence. The measuring lights were modulated with alternating 10-μs pulses at a repetition rate of 1200 Hz. The PhytoWin software was applied for analyzing the primary information in signals from the independent fluorescence. After that, the calculated total chlorophyll content was further divided by the algal concentration to obtain the chlorophyll content per cell (×10^3^ μg/L/cell).

### 5.4. Co-Culture Experiment at Stationary Phase and Decline Phase

In addition to the study of bacterial challenge to algal growth in the log phase, the strain *Bacillus anthracis* was selected to evaluate the effects on algal growth at the stationary phase and the decline phase. The 1022M2C12 in the early stationary and decline phase was applied for the co-culture experiment, respectively. For the stationary phase co-culture experiment, 5 × 10^5^ cells/mL and additional higher final concentrations of 1 × 10^6^ cells/mL and 2 × 10^6^ cells/mL of bacteria were added. The concentration of 1022M2C12 was monitored every three days in triplicate by an optical microscope. For the decline phase of the co-culture experiment, 5 × 10^5^ cells/mL of bacteria suspension was added when the algae entered the decline phase. Besides, to simulate the effects of nutrient supplementation and secondary metabolite removal, additional reference experiments were performed by (1) supplementing 5 mL of 100× K medium stock solution into 500 mL of algal culture or (2) removing 480 mL of old medium and supplementing an equal volume of fresh K medium. During the manipulation, all of the algal cells settled to the bottom, and were not removed with the old medium.

### 5.5. Bioactive Substance Investigation

To evaluate the presence of potential bioactive substances from *B. anthracis*, aliquots of living bacterial suspension were ultrasonic treated to obtain the bacterial extracts and centrifuged by 12,000× *g* to obtain the supernatants. The 1022M2C12 in the early stationary phase (about 2500 cells/mL) was used for co-culture with the bacterial extracts and supernatants from different final concentrations of 5 × 10^5^ cells/mL, 1 × 10^6^ cells/mL, and 2 × 10^6^ cells/mL living bacterial suspension. To simulate nutrient supplementation effects, additional reference experiments were conducted by supplementing 5 mL of 100× K medium stock solution into the 500-mL algal culture.

### 5.6. Species-Specificity Survey

To survey the species-specific activity of *B. anthracis* on the growth of different strains of *Gambierdiscus*, additional co-culture experiments were performed using 1112M1M03 (*Gambierdiscus* sp. type 6, 0.02 pg P-CTX-1/cell) and 1021M1DC4 (*Gambierdiscus* sp. type 6, no toxicity). A final concentration of 5 × 10^5^ cells/mL of bacterial suspensions were co-cultured with the algal cultures. The algal concentration was monitored in triplicate using the same method as previously described.

### 5.7. Algal Toxicity Study

During the above described co-culture experiment of all of the AHL-producing bacteria with the 1022M2C12, the CTX toxins were also extracted from parallel experimental groups to evaluate the effects of QS bacteria on algal toxicity. The total algal cells in 500-mL culture medium were collected and extracted with MeOH under sonication. The extracts were evaporated in a rotary evaporator (N1200A, EYELA, Tokyo, Japan), and then reconstituted in MeOH and mixed with HPLC water and dichloromethane (DCM) in a separator funnel. After partitioning, the DCM extracts were evaporated and rinsed with MeOH. The toxicity of extracts was quantified by the Mouse Neuroblastoma Assay (MNA) [54], which measures the proliferation of Neuroblastoma cells (Neuro-2a) dosed with toxic extracts by MTT [3-(4,5-dimethyl-thiazol-2-yl)2,5-diphenyltetrazolium bromide] assay. The results were standardized by P-CTX-1 standard curves and reported as mean P-CTX-1 equivalents.

### 5.8. Statistical Analysis

For all of the experimental results, the mean and standard deviations from triplicate experiments were calculated by Microsoft Excel 2013 and Origin Lab 8.5. The Student’s *t*-test in SPSS 20.0 (IBM, New York, NY, USA, 2011) was used to detect significant differences in experiments. A *p* value < 0.05 was statistically significant.

## Figures and Tables

**Table 1:** Screened N-acyl homoserine lactone (AHL)-producing bacterial strains for co-culture experiments.

No. *	Description	Bit Score	E Value	Ident
1	*Vibrio* sp. WC141014 16S ribosomal RNA gene, partial sequence	1836	0	99%
2	*Alteromonas macleodii* strain KS62 16S ribosomal RNA gene, partial sequence	2567	0	99%
3	*Marinobacter hydrocarbonoclasticus* strain NIOSSD020#224 16S ribosomal RNA gene, partial sequence	2097	0	97%
4	*Thalassospira* sp. KU27D2 gene for 16S rRNA, partial sequence	1611	0	99%
5	*Pseudomonas aeruginosa* strain CFV 16S ribosomal RNA gene, partial sequence	1094	0	100%
6	*Vibrio maritimus* strain CR-IV-34 16S ribosomal RNA gene, partial sequence	2084	0	97%
7	*Providencia vermicola* strain NBA-2365 16S ribosomal RNA gene, partial sequence	1131	0	99%
8	*Marinobacterium stanieri* S30 contig00002, whole genome shotgun sequence	2615	0	99%
9	*Bacillus anthracis* strain C1E4 16S ribosomal RNA gene, partial sequence	1962	0	99%

* The GenBank accession number is KY777596-KY777604.

## Supplementary Materials

The following are available online at http://www.mdpi.com/2072-6651/10/7/257/s1, Figure S1: (**a**) The fast liquid AHL producers screening method in a 96-well microtiter plate and (**b**) the traditional solid agar plate screening results, with indicator strain A136. Black arrow: negative control (*E. coli* DH 5α); white arrow: blank control (water); red arrow: positive control (*P. aeruginosa* PAO1); green arrow: potential AHL-producing bacteria; 1 to 9: the screened AHL-producing strains, Figure S2: Taxonomic distribution at the genus level of the four sampling sites, Figure S3: Phylogenetic tree generated using the 16s rRNA gene of the screened AHL-producing bacterial species/phylotypes. Statistical method: Neighbor-joining. Number of bootstrap replications: 500. Model: Maximum Composite Likelihood, Table S1: Distribution of the corresponding genus of the screened QS bacteria, Table S2: *Gambierdiscus* strains from Marakei, Republic of Kiribati, Table S3: The gene fragment for the phylogenetic analyses, Table S4: Growth rate (divisions day^−1^) of 1022M2C12 in co-culture experiment.
