# Supplementary material for: Growth and Toxin Production of Gambierdiscus spp. Can Be Regulated by Quorum-Sensing Bacteria
Source: Toxins (Basel). 2018 Jun 22;10(7):257. doi: 10.3390/toxins10070257 (PMC6071102; doi:10.3390/toxins10070257)
Supplement: Supplementary file 1 [file toxins-10-00257-s001.pdf]

Supplementary Materials: Growth and Toxin Production of *Gambierdiscus* spp. Can be Regulated by Quorum-Sensing Bacteria

Bo Wang, Mimi Yao, Jin Zhou, Shangjin Tan, Hui Jin, Feng Zhang, Yim Ling Mak, Jiajun Wu, Leo Lai Chan and Zhonghua Cai

1. Experimental section

Calculation of algal growth rate

During the co-culture experiment, the growth rate (divisions day^{-1}) was calculated using data (n = 5) of algal concentration from the exponential portion of the growth curve by least squares regression [1] as follows:

$$\text{Growth rate} = \frac{n \times \sum^{n}(t_i \times lnC_i) - [\sum^{n}(t_i) \times \sum^{n}(lnC_i)]}{ln2 \times \{n \times \sum^{n}(t_i^2) - [\sum^{n}(t_i)]^2\}}$$

where t_i = co-culture day, C_i = algal concentration and n = sample number. The average growth rate was calculated in triplicates.

2. Figures and Tables

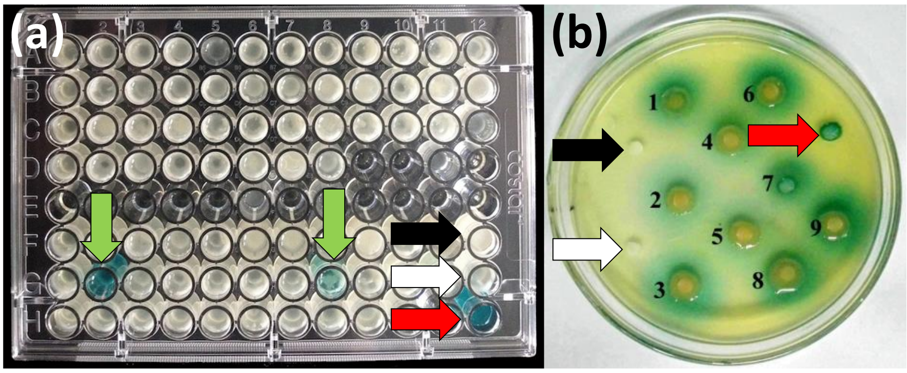

Figure S1. (a) The fast liquid AHL producers screening method in a 96-well microtiter plate and **(b)** the traditional solid agar plate screening results, with indicator strain A136. Black arrow: negative control (*E. coli* DH 5α); white arrow: blank control (water); red arrow: positive control (*P. aeruginosa* PAO1); green arrow: potential AHL-producing bacteria; 1 to 9: the screened AHL-producing strains.

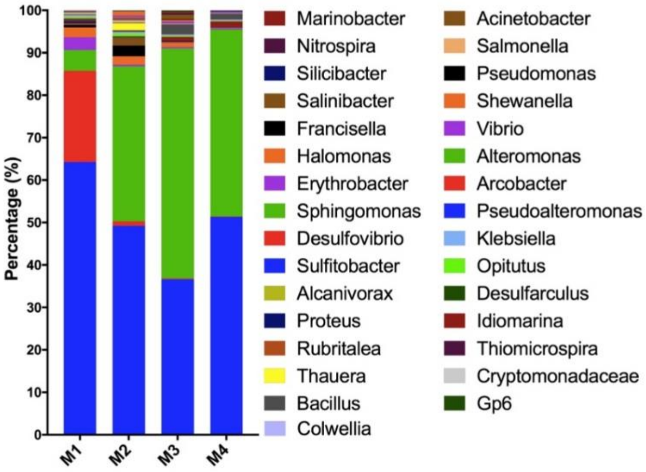

Figure S2. Taxonomic distribution at the genus level of the four sampling sites.

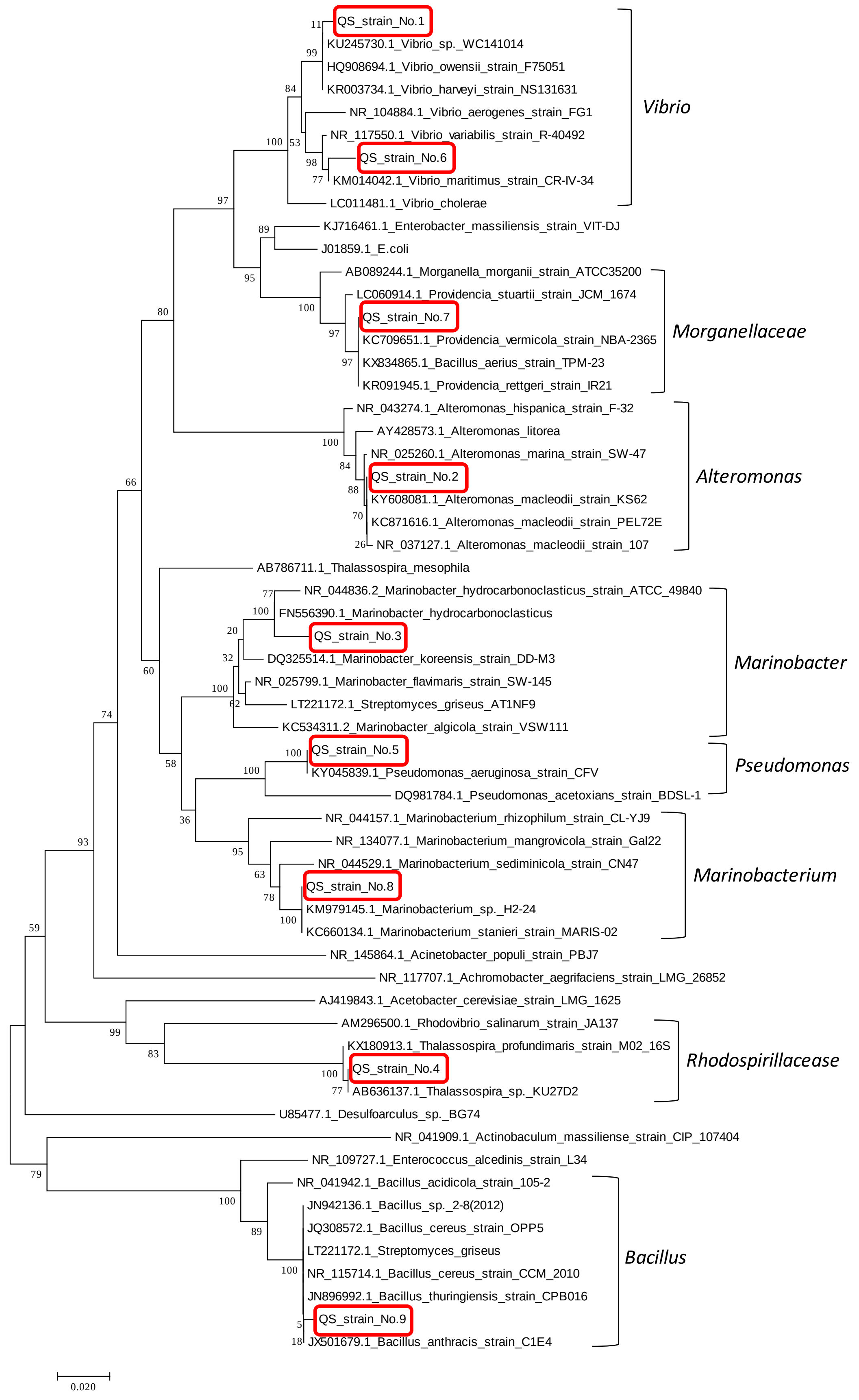

Figure S3. Phylogenetic tree generated using the 16s rRNA gene of the screened AHL-producing bacterial species/phylotypes. Statistical method: Neighbor-joining. Number of bootstrap replications: 500. Model: Maximum Composite Likelihood.

Table S1. Distribution of the corresponding genus of the screened QS bacteria.

Strains	Genus	Distribution of the genus (%)			
		M1	M2	M3	M4
Vibrio sp.	*Vibrio*	3.02	0.36	0.34	0.32
A. macleodii	*Alteromonas*	4.89	36.52	54.2	44.1
M. hydrocarbonoclasticus	*Marinobacter*	0	0	0.25	0.09
Thalassospira sp.	*Thalassospira*	-*	-	-	-
P. aeruginosa	*Pseudomonas*	0.54	2.55	0.03	0.11
V. maritimus	*Vibrio*	3.02	0.36	0.34	0.32
P. vermicola	*Providencia*	-	-	-	-
M. stanieri	*Marinobacterium*	0	0	0.25	0.09
B. anthracis	*Bacillus*	0.2	0.44	2.36	1.31

* Could not be detected at genus level.

Table S2. *Gambierdiscus* strains from Marakei, Republic of Kiribati.

Sample	Species name	Toxicity (pg P-CTX-1 eq cell^{-1})
1022M2C12	*Gambierdiscus* sp. type 5	0.004
1112M1M03	*Gambierdiscus* sp. type 6	0.02
1021M1DC4	*Gambierdiscus* sp. type 6	0

Table S3. The gene fragment for the phylogenetic analyses.

**>QS strain No.1**
ATGGCGGCAGCTACCATGCAGTCGAGCGGAACGAGTTATCTGAACCTTCGGGGGACGATAACGGC<br>GTCGAGCGGCGGACGGGTGAGTAATGCCTAGGAAATTGCCCTGATGTGGGGGATAACCATTGGAA<br>ACGATGGCTAATACCGCATAATGCCTACGGGCCAAAGAGGGGGACCTTCGGGCCTCTCGCGTCAGG<br>ATATGCCTAGGTGGGATTAGCTAGTTGGTGAGGTAATGGCTCACCAATGCGACGATCCCAAGCTGG<br>TCTGAGAGGATGATCAGCCACACTGGAACTGAGACACGGTCCAGACTCCTACGGGAGGCAGCAGT<br>GGGGAATATTGCACAATGGGCGCAAGCCTGATGCAGCCATGCCGCGTGTGTGAAGAAGGCCTTCG<br>GGTTGTAAAGCACTTTCAGTCGTGAGGAAGGTGGTGTAGTTAATAGCTGTATTATTTGACGTTAGCG<br>ACAGAAGAAGCACCGGCTAACTCCGTGCCAGCAGCCGCGGTAATACGGAGGGTGCGAGCGTTGAT<br>CGGAATTACTGGGCGTAAAGCGCATGCAGGTGGTTTGTTAAGTCAGATGTGAAAGCCCGGGGCTCA<br>ACCTCGGAATAGCATTTGAAACTGGCAGACTAGAGTACTGTAGAGGGGGGTAGAATTTCAGGTGTA<br>GCGGTGAAATGCGTAGAGATCTGAAGGAATACCGGTGGCGAAGGCGGCCCCCTGGACAGATACTG<br>ACACTCAGATGCGAAAGCGTGGGGAGCAAACAGGATTAGATACCCTGGTAGTCCACGCCGTAAAC<br>GATGTCTACTCGGAGGTTGTGGCCTTGAGGCGTGGCTTTCGGAGCTAACGCGTTAAGTAGACCGCCT<br>GGGGAGTACGGTCGCAAGATTAAAACTCAAATGAATTGACGGGGGCCCGCACAAGCGGTGGAGCA<br>TGTGGTTTAATTCGATGCAACGCGAAGAACCTTACCTACTCTTGACATCCAGAGAACTTTCCAGAGA<br>TGGATTGGTGCCTTCGGGAACTCTGAGAGAGGTGCTGCATGGCTGTCGTCAGCTCGTGTTGTGAAAT<br>GTTGGGTTAAGTCCCGCAACGAGCGCAACCCTTATCCTTGTCTGCCAGCGAGTAATGTCGGGAACTC<br>CAGGGAGACTGCCGGTGATAAACCGGAGGAAGGTGGGGACGACGTCAAGTCATCATGGCCCTTAC<br>GAGTAGGGCTACACACGTGCTACAATGGCGCATACAGAGGGCAGCAAGCTAGCGATAGTGAGCGA<br>ATCCCAAAAAGTGCGTCGTAGTCCGGATTGGAGTCTGCAACTCGACTCCATGAAGTCGGAATCGCT<br>AGTAATCGTGGATCAGAATGCCACGGTGAATACGTTCCCGGGCCTTGTACACACCGCCCGTCACAC<br>CATGGGAGTGGGCTGCAAAAGAAGTAGGTAGTTTAACCTTCGGGAGGACGCTACCACTTAGTCTGC
**>QS strain No.2**
ACATGCAAGTCGTACCAATAACATCATACTAGCTTGCTAGAAGATGACGAGTGGCGGACGGGTGA<br>GTAATGCTTGGGAACTTGCCTTTGCGAGGGGGATAACAGTTGGAAACGACTGCTAATACCGCATAA<br>TGTCTTCGGACCAAACGGGGCTTCGGCTCCGGCGCAAAGAGAGGCCCAAGTGAGATTAGCTAGTTG<br>GTAAGGTAACGGCTTACCAAGGCGACGATCTCTAGCTGTTCTGAGAGGAAGATCAGCCACACTGGG<br>ACTGAGACACGGCCCAGACTCCTACGGGAGGCAGCAGTGGGGAATATTGCACAATGGGGGAAACC<br>CTGATGCAGCCATGCCGCGTGTGTGAAGAAGGCCTTCGGGTTGTAAAGCACTTTCAGTTGTGAGGA<br>AAAGTTAGTAGTTAATACCTGCTAGCCGTGACGTTAACAACAGAAGAAGCACCGGCTAACTCCGTG<br>CCAGCAGCCGCGGTAATACGGAGGGTGCGAGCGTTAATCGGAATTACTGGGCGTAAAGCGCACGC<br>AGGCGGTTTGTTAAGCTAGATGTGAAAGCCCCGGGCTCAACCTGGGATGGTCATTTAGAACTGGCA

GACTAGAGTCTTGGAGAGGGGAGTGGAATTCCAGGTGTAGCGGTGAAATGCGTAGATATCTGGAG
GAACATCAGTGGCGAAGGCGACTCCCTGGCCAAAGACTGACGCTCATGTGCGAAAGTGTGGGTAG
CGAACAGGATTAGATACCCTGGTAGTCCACACCGTAAACGCTGTCTACTAGCTGTGTGTGTCTTTAA
GACGTGCGTAGCGAAGCTAACGCGCTAAGTAGACCGCCTGGGGAGTACGGCCGCAAGGTTAAAAC
TCAAATGAATTGACGGGGGCCCGCACAAGCGGTGGAGCATGTGGTTTAATTCGATGCAACGCGAA
GAACCTTACCTACACTTGACATGCTGAGAAGTTACTAGAGATAGTTTCGTGCCTTCGGGAACTCAGA
CACAGGTGCTGCATGGCTGTCGTCAGCTCGTGTCGTGAGATGTTGGGTTAAGTCCCGCAACGAGCG
CAACCCTTGTCCTTAGTTGCCAGCCTTAAGTTGGGCACTCTAAGGAGACTGCCGGTGACAAACCGG
AGGAAGGTGGGGACGACGTCAAGTCATCATGGCCCTTACGTGTAGGGCTACACACGTGCTACAATG
GCATTTACAGAGGGAAGCGAGACAGTGATGTGGAGCGGACCCCTTAAAGAATGTCGTAGTCCGGA
TTGGAGTCTGCAACTCGACTCCATGAAGTCGGAATCGCTAGTAATCGCAGGTCAGAATACTGCGGT
GAATACGTTCCCGGGCCTTGTACACACCGCCCGTCACACCATGGGAGTGGGATGCAAAAGAAGTA
GTTAGTCTAACCTTCGGGAGGACGATTACCACTT

>QS strain No.3

AAGCTTGCTTCCCGCTGACGAGCGGCGGACGGGTGAGTAATGCTTAGGAATCTGCCCAGTAGTGGG
GGATAGCCCGGGGAAACCCGGATTAATACCGCATACGTCCTACGGGAGAAAGCAGGGGATCTTCG
GACCTTGCGCTATTGGATGAGCCTAAGTCGGATTAGCTAGTTGGTGGGGTAAAGTCCTACCAAGGC
GACGATCCGTAGCTGGTCTGAGAGGATGATCAGCCACATCGGGACTGAGACACGGCCCGAACTCCT
ACGGGAGGCAGCAGTGGGGAATATTGGACAATGGGGGCGACGCTGATCCAGCCATGCCGCGTGTG
TGAAGAAGGCTTTCGGGTTGTAAAGCACTTTCAGTAGGGAGGAAAACTTTATGGTTAATACCCATG
AGGCTTGACGTTACCTACAGAAGAAGCACCGGCTAACTCCGTGCCAGCAGCCGCGGTAGTACGGA
GGGTGCTAGCGTTAATCGGAATTACTGGGCGTAAAGCGCGCGTAGGTGGTTTGGTGAGCGAGATGT
GAAAGCCCCGGGCTTAACCTGGGAACGGCATTTCGAACTGTCAGACTAGAGTGTGGTAGAGGGTA
GTGGAATTTCCTGTGTAGCGGTGAAATGCGTAGATATAGGAAGGAACACCAGTGGCGAAGGCGGC
TAGCCTGGACCAACCACTGATCACTGAGGTGCGAAAGCGTGGGGAGCAAACAGGATTAGATACCC
TGGTATTCCACGCCGTAAACGATGTCAACTAGCCGTTGGGACTCTTGAAGTCTTAGTGGCGCAGCTA
ACGCACTAAGTTGACCGCCTGGGGAGTACGGCCGCAAGGTTAAAACTCAAATGAATTGACGGGGG
CCCGCACAAGCGGTGGAGCATGTGGTTTAATTCGACGCAACGCGAAGAACCTTACCTGGCCTTGAC
ATCCAGAGAACTTTGCAGAGATGGGTTGGTGCCTTCGGGAACTCTGAGACAGGTGCTGCATGGCTG
TCGTCAGCTCGTGTCGTGAGATGTTGGGTTAAGTCCCGTAACGAGCGCAACCCCTATCCCTGGTTGC
TAGCAGGTAATGCTGAGAACTCCAGGGAGACTGCCGGTGACAAACCGGAGGAAGGTGGGGATGAC
GTCAGGTCATCATGGCCCTTACGGCCAGGGCTACACACGTGCTACAATGGCGCGTACAGAGGGCTG
CCAACTCGCGAGAGTGAGCCAATCCCTTAAAACGCGTCGTAGTCCGGATCGGAGTCTGCAACTCGA
CTCCGTGAAGTCGGAATCGCTAGTAATCGCGAATCAGAATGTCGCGGTGAATACGTTCCCGGGCCT
TGTACACACCGCCCGTCACACCATGGGAGTGGATTGAACCAGAAGTA

>QS strain No.4

GTTTGAGAGGATGATCAGCCACACTGGGACTGAGACACGGCCCAGACTCCTACGGGAGGCAGCAG
TGGGGAATATTGGACAATGGGGGCAACCCTGATCCAGCCATGCCGCGTGAGTGAAGAAGGCCTTC
GGGTTGTAAAGCTCTTTCAGATGCGAAGATGATGACGGTAACATCAGAAGAAGCCCCGGCTAATTT
CGTGCCAGCAGCCGCGGTAATACGAAAGGGGCTAGCGTTGTTCGGATTTACTGGGCGTAAAGGGCA
CGCAGGCGGTCTTGCCAGTCAGGGGTGAAAGCCCGGGGCTCAACCCCGGAACTGCCTCTGATACTG
CAAGACTAGAGACTAGGAGAGGGTGGTGGAATTCCCAGTGTAGAGGTGAAATTCGTAGATATTGG
GAGGAACACCAGAGGCGAAGGCGGCCACCTGGACTAGATCTGACGCTCAGGTGCGAAAGCGTGGG
GAGCAAACAGGATTAGATACCCTGGTAGTCCACGCCGTAAACGATGAGTGCTAGTTGTCGGGACTT
CGATTTCGGTGACGCAGCTAACGCATTAAGCACTCCGCCTGGGGAGTACGGTCGCAAGATTAAAAC
TCAAAGGAATTGACGGGGGCCCGCACAAGCGGTGGAGCATGTGGTTTAATTCGAAGCAACGCGCA
GAACCTTACCAACCCTTGACATCCCTATCGCGATTTCCAGAGATGGATATCATCAGTTCGGCTGGAT
AGGTGACAGGTGCTGCATGGCTGTCGTCAGCTCGTGTCGTGAGATGTTGGGTTAAGTCCCGCACGA
GCGCAACCCCTGTTCCTAGTGCCAGCATTTAGTTGGGCACTCTAGGAGACTGCCGTGACAAGCCGG
AGGAAGCCGGGGATGACGTCAAGTCCTCATGGCC

>QS strain No.5

TGGGAGGAAGGGCAGTAAGTTAATACCTTGCTGTTTTGACGTTACCAACAGAATAAGCACCGGCTA
ACTTCGTGCCAGCAGCCGCGGTAATACGAAGGGTGCAAGCGTTAATCGGAATTACTGGGCGTAAAG
CGCGCGTAGGTGGTTCAGCAAGTTGGATGTGAAATCCCCGGGCTCAACCTGGGAACTGCATCCAAA
ACTACTGAGCTAGAGTACGGTAGAGGGTGGTGGAATTTCCTGTGTAGCGGTGAAATGCGTAGATAT
AGGAAGGAACACCAGTGGCGAAGGCGACCACCTGGACTGATACTGACACTGAGGTGCGAAAGCGT
GGGGAGCAAACAGGATTAGATACCCTGGTAGTCCACGCCGTAAACGATGTCGACTAGCCGTTGGG
ATCCTTGAGATCTTAGTGGCGCAGCTAACGCGATAAGTCGACCGCCTGGGGAGTACGGCCGCAAGG

TTAAAACTCAAATGAATTGACGGGGGCCCGCACAAGCGGTGGAGCATGTGGTTTAATTCGAAGCAA
CGCGAAGAACCTTACCTGGCCTTGACATGCTGAGAACTTTCCAGAGATGGATTGGTGCCTTCGGGA

>QS strain No.6

GTGAGTAATGCCTGGGAAATTGCCCTGATGTGGGGGATAACCATTGGAAACGATGGCTAATACCGC
ATAATAGCTTCGGCTCAAAGAGGGGGACCTTCGGGCCTCTCGCGTCAGGATATGCCCAGGTGGGAT
TAGCTAGTTGGTGAGGTAATGGCTCACCAAGGCGACGATCCCTAGCTGGTCTGAGAGGATGATCAG
CCACACTGGAACTGAGACACGGTCCAGACTCCTACGGGCGGCACCAGTGGCGAATATTGCACAAT
GGGCGCAAGCCTGATGCAGCCATGCCGCGTGTATGAATAAGGCCTTCGGGTTGTAAAGTACTTTCA
GCAGTGAGGAAGGTGGTGTCGTTAATAGCGGCATCATTTGATGTTAGCTGCAGAAGAAGCACCGGC
TAACTCCGTGCCAGCAGCCGCGGTAATACGGAGGGTGCGAGCGTGGATCGGAATTACTGGGCGCA
AAGCGCATGCAGGTGGTTAGTTAAGTCAGATGTGAAAGCCCGGGGCTCAACCTCGGAACTGCATTT
GAAACTGGCTGACTAGAGTACTGTAGAGGGGGGTAGAATTTCAGGTGTAGCGGTTAAATGCGTAGA
GATCTGAAGGAATACCGGTGGCGAAGGCGGCCCCCTGGACAGATACTGACACTCAGATGCGAAAG
CGTGGGGAGCACACAGGATTAGATACCCTGGTAGTCCACGCCGTAAACGATGTCTACTTGGAGGTT
GTGGCCTTGAGCCGTGGCTTTCGGAGCTAACGCGTTAAGTAGACCGCCTGGGGAGTACGGTCGCAA
GATTAAAACTCAAATGAATTGACGGGGGCCCGCACAAGCGGTGGAGCATGTGGTTTAATTCGATGC
AACGCGAAGAACCTTACCTACTCTTGACATCCAGAGAACTTAGCAGAGATGCTTTGGTGCCTTCGG
GAACTCTGAGACAGGTGCTGCATGGCTGTCGTCAGCTCGTGTTGTGAAATGTTGGGTTAAGTCCCGC
AACGAGCGCAACCCTTATCCTTGTTTGCCAGCACTTCGGGTGGGAACTCCAGGGAGACTGCCGGTG
ATAAACCGGAGGAAGGTGGGGACGACGTCAAGTCATCATGGCCCTTACGAGTAGGGCTACACACG
TGCTACAATGGCGCATACAGAGGGCRGCCAACTTGCGAAAGTGAGCGAATCCCAAAAAGTGCGTC
GTAGTCCGGATTGGAGTCTGCAACTCGACTCCATGAAGTCGGAATCGCTAGTAATCGTGGATCAGA
ATGCCACGGTGAATACGTTCCCGGGCCTTGTACACACCGCCCGTCACACCATGGGAGTGGGCTGCA
AAAGAAGTGGGTAGTTTAACCTTCGGGAGGACGCTCACCACTTTGTGGTTCATGACTGGGGTGAAG
TCGTAACAAGGTA

>QS strain No.7

AGGCCCTAGGGTTGTAAAGTACTTTCAGTCGGGAGGAAGGCGTTGTTGCTAATATCAACAACGATT
GACGTTACCGACAGAAGAAGCACCGGCTAACTCCGTGCCAGCAGCCGCGGTAATACGGAGGGTGC
AAGCGTTAATCGGAATTACTGGGCGTAAAGCGCACGCAGGCGGTTGATTAAGTTAGATGTGAAATC
CCCGGGCTTAACCTGGGAATGGCATCTAAGACTGGTCAGCTAGAGTCTTGTAGAGGGGGGTAGAAT
TCCATGTGTAGCGGTGAAATGCGTAGAGATGTGGAGGAATACCGGTGGCGAAGGCGGCCCCCTGG
ACAAAGACTGACGCTCAGGTGCGAAAGCGTGGGGAGCAAACAGGATTAGATACCCTGGTAGTCCA
CGCTGTAAACGATGTCGATTTGAAGGTTGTTCCCTTGAGGAGTGGCTTTCGGAGCTAACGCGTTAAA
TCGACCGCCTGGGGAGTACGGCCGCAAGGTTAAAACTCAAATGAATTGACGGGGCCCGCACAAGC
GGTGGAGCATGTGGTTTAATTCGATGCAACGCGAAGAACCTTACCTACTCTTGACATCCAGAGAAC
TTAGCAGAGATGCTTTAGTGCCTTCGG

>QS strain No.8

CGTGGCGGCAGCCTACACATGCAGTCGAGCGGTAACAGAACTAGCTTGCTAGTTGCTGACGAGCGG
CGGACGGGTGAGTAACGCGTGGGAATCTGCCTGGTAGTGGGGGACAACAGTTGGAAACGACTGCT
AATACCGCATACGCCCTTCGGGGGAAAGTAGGGGATCTTCGGACCTTACGCTATCAGATGAGCCCG
CGTCGGATTAGCTTGTTGGTGAGGTAAAGGCTCACCAAGGCGATGATCCGTAGCTGGTCTGAGAGG
ATGATCAGCCACACTGGGACTGAGACACGGCCCAGACTCCTACGGGAGGCAGCAGTGGGGAATAT
TGGACAATGGGGGCAACCCTGATCCAGCCATGCCGCGTGTGTGAAGAAGGCCTTCGGGTTGTAAAG
CACTTTCAGATGTGAGGAAAGGTGTGTGGTTAATACCCGCATACTGTGACGTTAACATCAGAAGAA
GCACCGGCTAACTCCGTGCCAGCAGCCGCGGTAATACGGAGGGTGCAAGCGTTAATCGGAATTACT
GGGCGTAAAGCGCACGTAGGTGGTTTGGTCAGTCAGATGTGAAAGCCCCGGGCTCAACCTGGGAA
CTGCACCTGATACTGCCAGACTAGAGTACGGTAGAGGGTAGTGGAATTTCCGGTGTAGCGGTGAAA
TGCGTAGAGATCGGAAGGAACACCAGTGGCGAAGGCGACTGCCTGGACCGATACTGACACTGAGG
TGCGAAAGCGTGGGTAGCAAACAGGATTAGATACCCTGGTAGTCCACGCCGTAAACGATGTCAACT
AGCCGTTGGGGGACTTGATCCTTTAGTGGCGCAGCTAACGCGATAAGTTGACCGCCTGGGGAGTAC
GGCCGCAAGGTTAAAACTCAAATGAATTGACGGGGGCCCGCACAAGCGGTGGAGCATGTGGTTTA
ATTCGAAGCAACGCGAAGAACCTTACCTACTCTTGACATCCTGCGAACTTGCTAGAGATAGCTTGGT
GCCTTCGGGAACGCAGTGACAGGTGCTGCATGGCTGTCGTCAGCTCGTGTTGTGAAATGTTGGGTTA
AGTCCCGTAACGAGCGCAACCCCTATCCTTATTTGCCAGCACTTCGGGTGGGAACTCTAAGGAGAC
TGCCGGTGACAAACCGGAGGAAGGTGGGGATGACGTCAAGTCATCATGGCCCTTACGAGTAGGGC
TACACACGTGCTACAATGGCCGGTACAGAGGGCCGCGAACTCGCGAGGGTAAGCAAATCTCAGAA
AACCGGTCGTAGTCCGGATCGCAGTCTGCAACTCGACTGCGTGAAGTCGGAATCGCTAGTAATCGC

GAATCAGAATGTCGCGGTGAATACGTTCCCGGGCCTTGTACACACCGCCCGTCACACCATGGGAGT
GGGTTGCACCAGAAGTAGCTAGTCTAACCTTCGGGAGGACGGTACCACGGGGGTTACAA

>QS strain No.9

CATGGCCGCGTGCTATACATGCAAGTCGAGCGAATGGATTAAGAGCTTGCTCTTATGAAGTTAGCG
GCGGACGGGTGAGTAACACGTGGGTAACCTGCCCATAAGACTGGGATAACTCCGGGAAACCGGGG
CTAATACCGGATAACATTTTGAACCGCATGGTTCGAAATTGAAAGGCGGCTTCGGCTGTCACTTATG
GATGGACCCGCGTCGCATTAGCTAGTTGGTGAGGTAACGGCTCACCAAGGCAACGATGCGTAGCCG
ACCTGAGAGGGTGATCGGCCACACTGGGACTGAGACACGGCCCAGACTCCTACGGGAGGCAGCAG
TAGGGAATCTTCCGCAATGGACGAAAGTCTGACGGAGCAACGCCGCGTGAGTGATGAAGGCTTTCG
GGTCGTAAAACTCTGTTGTTAGGGAAGAACAAGTGCTAGTTGAATAAGCTGGCACCTTGACGGTAC
CTAACCAGAAAGCCACGGCTAACTACGTGCCAGCAGCCGCGGTAATACGTAGGTGGCAAGCGTTA
TCCGGAATTATTGGGCGTAAAGCGCGCGCAGGTGGTTTCTTAAGTCTGATGTGAAAGCCCACGGCT
CAACCGTGGAGGGTCATTGGAAACTGGGAGACTTGAGTGCAGAAGAGGAAAGTGGAATTCCATGT
GTAGCGGTGAAATGCGTAGAGATATGGAGGAACACCAGTGGCGAAGGCGACTTTCTGGTCTGTAAC
TGACACTGAGGCGCGAAAGCGTGGGGAGCAAACAGGATTAGATACCCTGGTAGTCCACGCCGTAA
ACGATGAGTGCTAAGTGTTAGAGGGTTTCCGCCCTTTAGTGCTGAAGTTAACGCATTAAGCACTCCG
CCTGGGGAGTACGGCCGCAAGGCTGAAACTCAAAGGAATTGACGGGGGCCCGCACAAGCGGTGGA
GCATGTGGTTTATTCGAAGCAACGCGAGAACCTTACCAGGTCTCGACATCCTTCTGACAACCCTAG
AGATAGGGCTTCTTCTTCGGGAGCAGAGTTGACAGGTGATGCATGTTGTCGTCACTCGTGTCTGAGA
TGTTGGGTAAGTTCCGCAACGACGCAACCCGTGATCTTAGTTGCCTTCATTAAGTTGGG

Table S4. Growth rate (divisions day^{-1}) of 1022M2C12 in co-culture experiment.

Sample	Low concentration (5×10^3 cells/mL)	Medium concentration (5×10^4 cells/mL)	High concentration (5×10^5 cells/mL)
Bacteria No.1	0.347 ± 0.012	0.358 ± 0.008	0.326 ± 0.002
Bacteria No.2	0.335 ± 0.010	0.329 ± 0.018	0.332 ± 0.029
Bacteria No.3	0.351 ± 0.003	0.349 ± 0.004	0.056 ± 0.006
Bacteria No.4	0.326 ± 0.009	0.362 ± 0.009	0.349 ± 0.007
Bacteria No.5	0.338 ± 0.013	0.337 ± 0.004	0.350 ± 0.007
Bacteria No.6	0.356 ± 0.017	0.359 ± 0.004	0.394 ± 0.011
Bacteria No.7	0.367 ± 0.019	0.368 ± 0.010	0.381 ± 0.042
Bacteria No.8	0.389 ± 0.013	0.371 ± 0.004	0.394 ± 0.033
Bacteria No.9	0.335 ± 0.009	0.334 ± 0.010	0.284 ± 0.004
Control	0.369 ± 0.012	-	-

References

1. Yamaguchi, M.; Honjo, T. Effects of temperature, salinity and irradiance on the growth of the noxious red tide flagellate *Gymnodinium nagasakiense* (Dinophyceae). *Bull. Jpn. Soc. Sci. Fish. (Japan)* **1989**, *55*, 2029–2036.

© 2018 by the authors. Submitted for possible open access publication under the terms and conditions of the Creative Commons Attribution (CC BY) license (http://creativecommons.org/licenses/by/4.0/).
